# Optimizing the releasing strategy used for the biological control of the sugarcane borer *Diatraea saccharalis* by *Trichogramma galloi* with computer modeling and simulation

**DOI:** 10.1038/s41598-024-60146-y

**Published:** 2024-04-25

**Authors:** Adriano Gomes Garcia, Eric Wajnberg, José Roberto Postali Parra

**Affiliations:** 1 Department of Entomology and Acarology, São Paulo University/ ESALQ, Pádua Dias Avenue 11, Piracicaba, 13418-900 Brazil; 2 Inrae, 400 Route des Chappes, BP 167, 06903 Sophia Antipolis Cedex, France; 3 Inria, Projet Hephaistos, 06902 Sophia Antipolis, France

**Keywords:** Augmentative biological control, Ecological modeling, Monte Carlo simulations, Computational models, Ecological modelling

## Abstract

One of the challenges in augmentative biological control programs is the definition of releasing strategy for natural enemies, especially when macro-organisms are involved. Important information about the density of insects to be released and frequency of releases usually requires a great number of experiments, which implies time and space that are not always readily available. In order to provide science-based responses for these questions, computational models offer an in silico option to simulate different biocontrol agent releasing scenarios. This allows decision-makers to focus their efforts to more feasible options. The major insect pest in sugarcane crops is the sugarcane borer *Diatraea saccharalis*, which can be managed using the egg parasitoid *Trichogramma galloi*. The current strategy consists in releasing 50,000 insects per hectare for each release, in three weekly releases. Here, we present a simulation model to check whether this releasing strategy is optimal against the sugarcane borer. A sensitive analysis revealed that the population of the pest is more affected by the number of releases rather than by the density of parasitoids released. Only the number of releases demonstrated an ability to drive the population curve of the pest towards a negative growth. For example, releasing a total of 600,000 insects per hectare in three releases led to a lower pest control efficacy that releasing only 250,000 insects per hectare in five releases. A higher number of releases covers a wider range of time, increasing the likelihood of releasing parasitoids at the correct time given that the egg stage is short. Based on these results, it is suggested that, if modifications to the releasing strategy are desired, increasing the number of releases from 3 to 5 at weekly intervals is most likely preferable.

## Introduction

The use of parasitoid or predator insects in augmentative biological control, i.e., release of biological agents against insect pests in agriculture environments, has grown worldwide in different crops^1–3^. From a large variety of macro-organisms, including nematodes or parasitic wasps, biocontrol companies have strongly increased profit over the years (Forbes^4^). Efficient biological control programs are not easily implemented, given that a multidisciplinary effort is needed to cover all the necessary steps, involving different activities, e.g., taxonomic identification, selectivity testing and transference of the methods to the producers. However, they are still cheaper and more environmental-friendly than the use of agrochemicals^5^.

Whereas biological control generally seems to work satisfactorily into the context of Integrated Pest Management (IPM), this expansion at fast pace has led to several questions that still remain unsolved. What is the optimized number of biocontrol agents to be released? What is the optimal interval between releases? What is the importance of abiotic factors? What is the efficacy of the biological control agent under different releasing and/or agro-ecological conditions? Unfortunately, for some, if not most of these questions, it is difficult to provide a concrete response, given the time and resources that are often scarce. In this context, computational models are a good option to simulate different scenarios involving crop systems^6^.

Computational models can allow one to explore scenarios and conditions that would be difficult to study with experiments due to time length or lack of space. This can be useful in many different ways, including hypothesis testing, generating new insights, suggesting and interpreting experiments, tracing chains of causation, doing sensitivity analyses, integrating knowledge, and inspiring new approaches^7–9^.

An interesting approach combines biological control with optimization processes and some previous works using this approach are noteworthy. Developing an individual-based model which included stochasticity and spatial structure, van Roermund et al.^10^ simulated the biological control of the greenhouse whitefly with the parasitoid *Encarsia formosa* (Gahan) (Hymenoptera: Aphelinidae). The authors identified the most important parasitoid parameters that influenced the level of control. Weber et al.^11^ developed a complex computational model based on a two-dimensional grid and Monte Carlo simulations to model the augmentative biological control against *Euschistus heros* (Fabricius) (Heteroptera: Pentatomidae) using *Telenomus podisi* (Ashmead) (Hymenoptera: Scelionidae). The authors tested different features in their simulations, e.g., number of parasitoids released, release of parasitoids in strips or points, spacing between points or strips, etc. Plouvier and Wajnberg^12^ identified a lack of cost-effective perspective in host-parasitoid models, developing a general individual-based model simulating the release of biocontrol agents for short-term control (inundative releases). In their model, the authors took into account the revenues and costs for the biocontrol practitioner that could be adapted for a wide range of systems.

The optimization of protocols using computational models can open perspectives to improve significantly the biological control of key insect pests. In this respect, *Diatraea saccharalis* (Fabricius) (Lepidoptera: Crambidae) is a key insect pest of sugarcane and other grasses^13,14^. that occurs from Florida to southern Argentina. This species has been well-studied regarding its biology, hosts and natural enemies^15^. Its larvae create galleries into the sugarcane stalks, leading to considerable losses. For its management, the parasitoid *Trichogramma galloi* (Zucchi) (Hymenoptera: Trichogrammatidae) appears to be an efficient biological control agent and is released in sugarcane fields in Brazil^5^. This parasitoid wasp parasitizes and kills eggs in the field, thus interrupting the embryonic development of *D. saccharalis*. In a few days, new wasps emerge from these eggs^16^. The current releasing strategy for biological control using this wasp species consists of three parasitoid releases at a density of 50,000 parasitoids per hectare, with a one-week interval. However, there is little scientific evidence that such a releasing strategy is the best to be adopted.

In the current work, we present a simulation model to optimize the release of *T. galloi* against *D. saccharalis*, analyzing the effect of release rate and the density of parasitoids per release. The model follows a similar structure proposed by Weber et al.^11^. The main question we want to address here is how to improve the pest control efficacy by altering the current releasing strategy and to identify the sensitivity of the management to each of the tested variables.

## Methodology

The model consists of a Monte Carlo simulation developed in C programming language. It investigates the population dynamics of the insect pest and the parasitoid using a 1000 × 1000 grid (1 cell = 1 × 1 m^2^), which represented a total area of 100 ha, a size large enough to prevent the insects to reach the border over the simulation. Each time step in the simulations represents 1 day. The dynamics of the pest and the parasitoid were simulated according to the description provided below as can be visualized in Supplementary Fig. S1.

### Insect development

The model was calibrated to represent the system *Diatraea saccharalis*-*Trichogramma galloi*. Therefore, we mimic a holometabolous life cycle for the pest, composed by egg, larval, pupal and adult stages. The development period of each stage was determined by the accumulated amount of energy at each day given by the difference between the environmental mean temperature and the lower development threshold, i.e., $T - T_{T}$^17^. Insects molt from a stage to another, when the accumulated amount of energy is equal to $K_{stage}$ which is the thermal constant for a particular stage of the species. We prioritize temperature-dependence associated with maturation delays in the code rather than for other rates because this parameter is the key variable determining the length of the cycle, impacting directly the releasing time, and its effect on insect development is well-known for the species considered in the studied system. In the simulations, we adopted a mean temperature equal to 30 °C as the model describes a situation at this constant temperature. The dynamics of the parasitoid followed the same structure, except that there are only two stages: immature (developed inside the parasitized host eggs) and adult.

### Longevity

We assumed that the longevity of adults at each age $\alpha$ followed a Normal distribution with $\mu$ and $\delta$ as mean and standard deviation of the total life span observed for adult insects, respectively.

### Oviposition

We assumed that insects lay a number of eggs $o_{a}$ per day that follows an exponential distribution, where $\lambda$ = total number of eggs per female, $\alpha$ = age of the adult female and $\eta$ is a constant defined according to data about the oviposition rhythm of the species obtained from previous published experiments^18^.

1 $$o_{\alpha } = \lambda \left[ {e^{ - \eta \alpha } - e^{{ - \eta \left( {\alpha + 1} \right)}} } \right]$$

### Initial condition

Simulations were initialized with four adult females of *D. saccharalis* at the center of the grid. Parasitoids were released only when the adult population reached a threshold $\tau$. Parasitoids are then released in an area located in the center of the grid corresponding to the area where the first eggs of *D. saccharalis* are reported in the simulation, at different densities depending on the simulation performed (see section *Simulation and analysis*).

### Movement

Two types of movement were considered: with or without preferred direction for both parasitoids and pests. Given that we were considering a plant host distributed homogeneously in the area, we assumed a movement without preferred direction for the insect pest. For this, we use the probability $D$ of reaching a particular distance $d$ given by an exponential distribution, with parameter $\beta$ based on the previous knowledge on the insect distribution^19^:

2 $$D_{d} = e^{{{ - \beta d}}}$$

Regarding the parasitoids, we assumed a preferred direction movement since parasitoids forage for hosts in the agricultural landscape and are known to respond to cues originating from their hosts^11^. For this, the parasitoids were assumed to examine their action radius centered on their current location and the probability of moving to a particular grid cell will depend on the relative density of hosts within this radius. If there is no host, the parasitoids follow a movement without preferred direction according to Eq. (2). We also included a daily probability $\varphi$ of a new infestation by the insect pest to occur in the grid (simulating the arrival of insects from outside of the area).

### Parametrization

**Figure 1:**
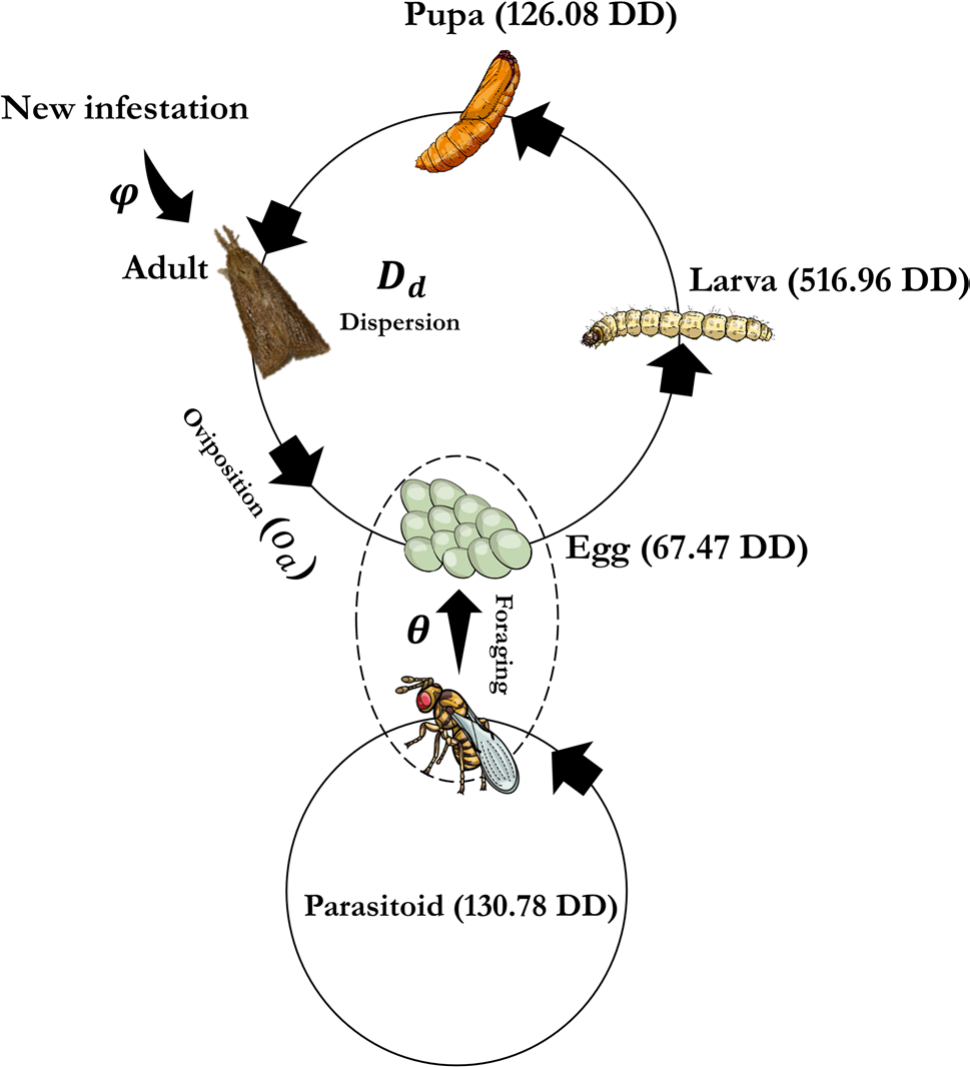
Schematization of the model structure. In order to molt to another stage, the pest needs to accumulate an amount of energy in degree-days (DD). The egg stage is the shortest insect stage. The parameter $o_{a}$ determines the number of new individuals produced by *D. saccharalis* females. The parameters $\theta$ and $\varphi$ correspond to the number of eggs parasitized by a parasitoid female and the probability of a new infestation by *D. saccharalis* in the simulation grid, respectively. Each adult have a probability $D$ of reaching a particular distance $d$ given by an exponential distribution.

The value assumed for each parameter defined in the model is presented in Tables 1 and 2. The model framework is shown in Fig. 1.

**Table 1:** List of model parameters for *Diatraea saccharalis* used as input in the computer program with the respective sources (DD: degree-days).

Parameter	Unit	Value	Source
Thermal constant ($K_{1}$) egg stage	DD	67.47	Melo and Parra^18^
Thermal constant ($K_{2}$) larval stage	DD	516.96	Melo and Parra^18^
Thermal constant ($K_{3}$) pupal stage	DD	126.08	Melo and Parra^18^
Lower development threshold ($T_{T1}$): egg	°C	11.2	Melo and Parra^18^
Lower development threshold ($T_{T2}$): larva	°C	7.3	Melo and Parra^18^
Lower development threshold ($T_{T3}$): pupa	°C	10.6	Melo and Parra^18^
Adult longevity (μ ± δ)	days	8.60 ± 0.95	Melo and Parra^18^
Pre-oviposition period (days)	days	2.16	Melo and Parra^18^
Sex ratio (proportion of females)		0.46	Melo and Parra^18^
Maximum distance travelled per day	meters	50	Caixeta^19^
β (exponential parameter)		0.2	Caixeta^19^
Net reproductive rate		84	Melo and Parra^18^
φ (probability of new infestation)		0.01

**Table 2:** List of model parameters for *Trichogramma galloi* used as input in the computer program with the respective sources (DD: degree-days).

Parameter	Unit	Value	Source
Thermal constant ($KI$)	DD	130.98	Cônsoli and Parra^16^
Lower development threshold ($T_{TI}$)	°C	13.6	Cônsoli and Parra^16^
Adult longevity(μ ± δ)	days	8.9 ± 0.7	Cônsoli and Parra^16^
Parasitized eggs per female (θ)	eggs/female	57	Cônsoli and Parra^16^
Offspring per egg parasitized	insect/egg	2	Cônsoli and Parra^16^
Action radius	meters	10	Broglio-Micheletti et al.^20^
Sex ratio (proportion females)		0.89	Cônsoli and Parra^16^

### Simulations and analysis

The current strategy for the release of *T galloi* against *D. saccharalis* consists of three releases, each with 50,000 parasitoids per hectare for each release at weekly intervals. We conducted simulations proposing changes in this protocol in order to quantify the effect of increasing insect densities and number of releases on the population dynamics of the insect pest in each case. We conducted simulations by doubling the density of parasitoids from 50,000 to 100,000 ha^−1^ and then from 50,000 to 200,000 ha^−1^ per event (each release), while keeping the release frequency constant. We also simulated an increase in the frequency of releases from three to four and from three to five, keeping the total number of released parasitoids constant at 50,000 parasitoids ha^−1^ per release event when varying the frequency, following weekly interval. We run each simulation over 150 time steps (days) because this period of time covers the maturation of the sugarcane plant and is sufficient to represent the entire period of action of the parasitoid before its effects begin to diminish.

A sensitivity analysis was performed to test the effect of each variable on the system outputs. Basically, we analyzed the effects of each particular variable of interest by varying it and fixing the values of all others, pinpointing the most important model parameters^21^. The goal here was to identify the key elements that can be modified in order to maximize the effect of a particular natural enemy^22–24^. Each simulation was replicated 20 times and averages with the 95% confidence interval were estimated to take into account the variability associated to stochastic components (e.g., parasitoid movement and probability of a new infestation). Increasing the number of replicates mechanically reduces standard errors, thereby consistently yielding significant results without the need for statistical comparison analyses. Hence, no statistical comparison was done on the results obtained. We also calculated the ratio $N$ between the simulated host larval population obtained in the modified protocol and in the standard protocol over time.

## Results

**Figure 2:**
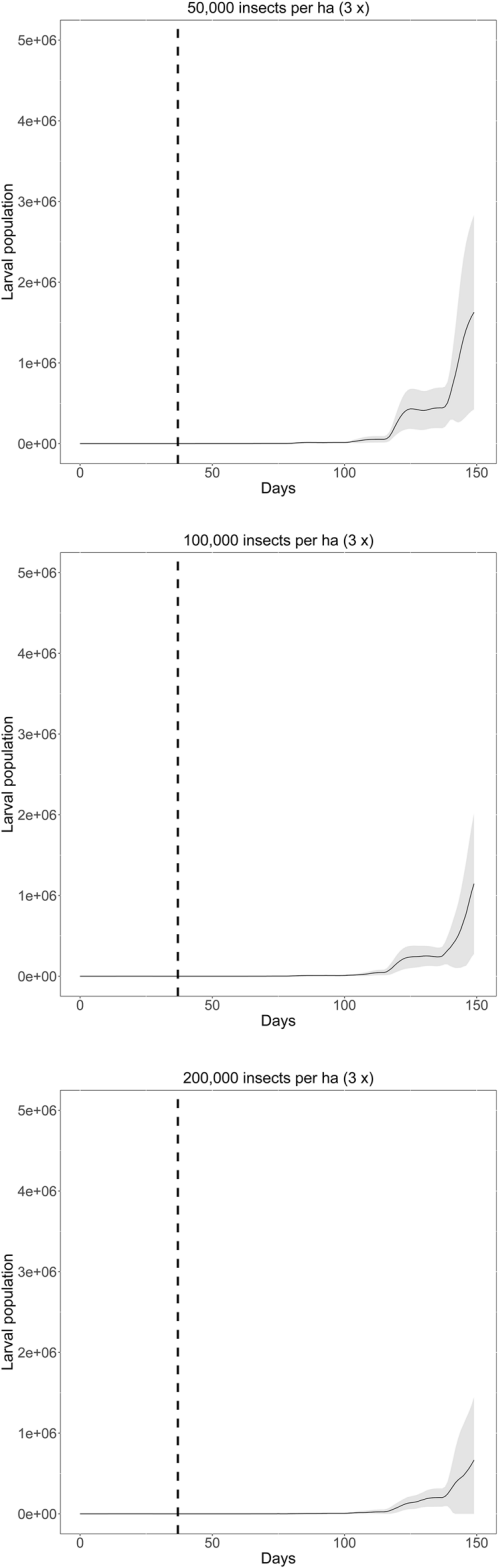
Dynamics of larval pest population over 150 days when different number of parasitoids are released, maintaining three releases at weekly intervals. The vertical dashed black line indicates the moment of the first release (day 36). The line indicates the mean curve of the 20 simulations and the shaded area corresponds to the 95% confidence interval.

Fixing the frequency of releases and varying the number of parasitoids released, we were able to identify changes in host population density, but not in the shape of the population curve, which is always increasing (Fig. 2). Figure 4a illustrates the impact of increasing parasitoid density from 50,000 to 200,000 ha^−1^ on the host larval population. Increasing the parasitoid density from 50,000 to 200,000 ha^−1^ reduced the larval population by approximately three times.

**Figure 3:**
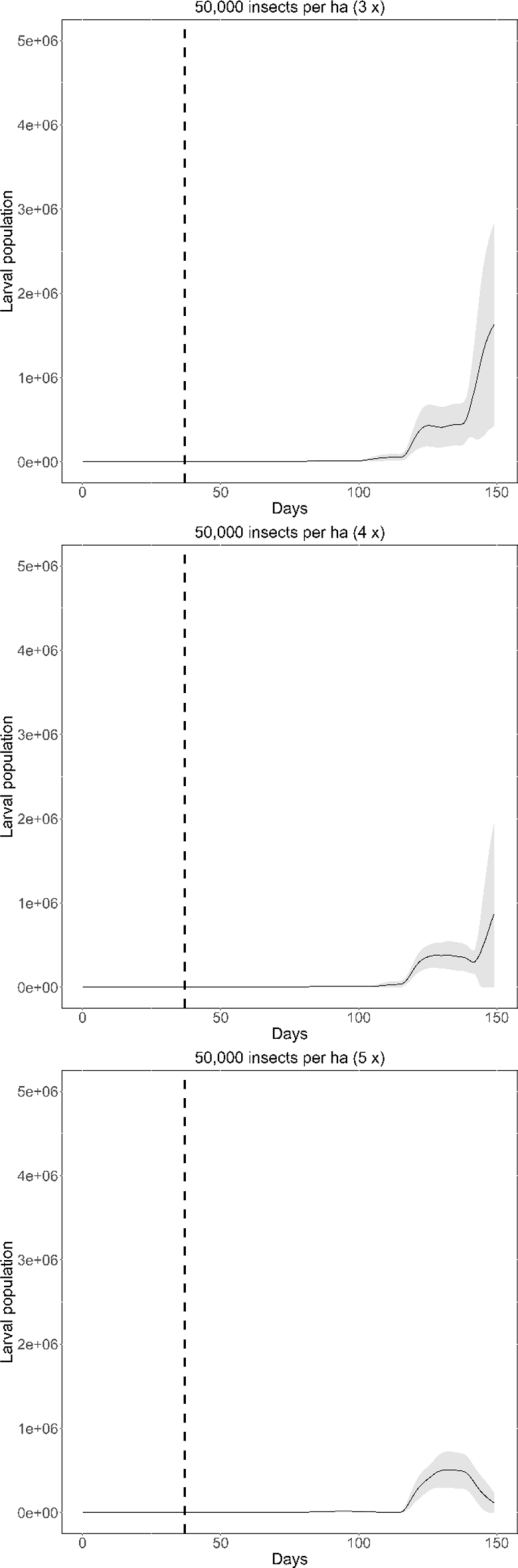
Dynamics of larval population over 150 days when the number of releases is changed, maintaining 50,000 parasitoids ha^−1^ for each release. The vertical dashed black line indicates the moment of the first release (day 36). The line indicates the mean curve of the 20 simulations and the shaded area corresponds to the 95% confidence interval.

**Figure 4:**
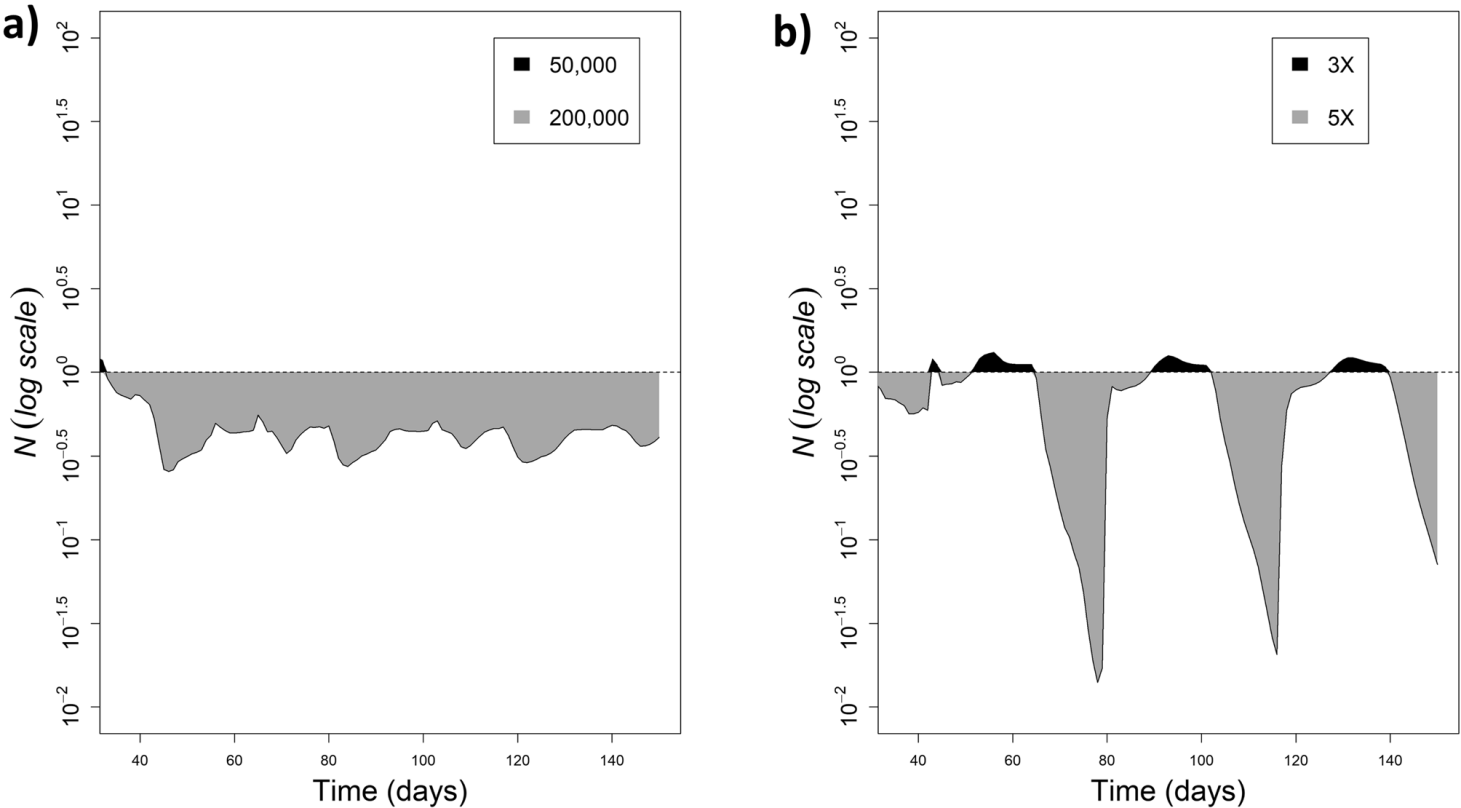
Different patterns observed for the ratio $N$ between (**a**) the current protocol and the scenario that increased parasitoid density from 50,000 to 200,000 and (**b**) the current protocol and the scenario that increased the release frequency from three to five only. The horizontal line corresponds to $N = 1$, which would be a condition when both protocols produce similar results. The filled areas indicates the protocol that was able to reduce more the larval population of the pest over the time and the magnitude of this reduction. The more the values are close to the dashed line the more the protocols are similar.

Fixing the parasitoid density and varying the number of releases only, we were able to identify changes both in the population density and in the shape of the population curve, which was reduced as the number of releases increased (Fig. 3). Figure 4b illustrates the impact of increasing the number of releases from three to five fixing the parasitoid density at 50,000 ha^−1^. It reduced the larval population by approximately 30 times.

## Discussion

The need to define clear protocols to release natural enemies in augmentative biological control programs is one of the main challenges in optimized pest control strategy. For the biological control of the sugarcane borer, we developed a simulation model that enabled us to identify that the key factor to modify the dynamics of the pest density is likely the number of releases since the model output appears to be more sensitive to this parameter, compared to the amount of parasitoids released. For example, releasing a total of 600,000 insects per hectare in three releases led to a lower pest control efficacy that releasing only 250,000 insects per hectare in five releases. This seems to indicate that, if some modification is desired in the biocontrol agent release protocol, it is preferable to increase the frequency of release. Therefore, our recommendation is to increase the frequency from three to five releases at weekly intervals, maintaining the parasitoid density at 50,000 parasitoids per hectare and per release.

A higher number of releases comprises a wider range of time, i.e., it maintains parasitoid populations active over a longer period of time, avoiding the insect pest to resurge, due to either the remaining population or the new migrant insect pests. Most works, including theoretical ones, target the optimization of augmentative biological control focusing on the number of parasitoids to be released per surface area^25,26^. However, the number of releases is likely also or even more important than the parasitoid density in the field, as a higher number of releases will increase the likelihood of releasing parasitoids at the correct time, when hosts are available^27,28^.

Futhermore, increasing the number of biological control agents released into an environment does not actually always increase the level of pest control^29^. Crowder^30^ found that, in 64% of the study cases involving natural enemies and targeted pests, a larger numbers of released natural enemies did not lead to a significant decrease in the density or an increase in the mortality of the pest. Our model indicated that this component appears to have a lower impact than the number of releases. A possible reason is that *T. galloi* is a parasitoid of eggs, which is the shortest stage of the host life^31^, therefore a higher number of releases increases the chances for natural enemies to find this proper stage.

It would be useful to have large time series data to observe these patterns in different situations and conditions in the field. However, unfortunately, on the contrary to laboratory studies which are well subsided scientifically (e.g.,^16^), there is still a lack of studies of the releasing strategy of *T. galloi* against *D. saccharalis*. In this respect, our model provides specific information about the studied system. Besides, differently from other models developed to study the system *D. saccharalis* and *T. galloi*^32^, we tried to reproduce aspects of the unpredictability nature of the biological processes through Monte Carlo simulations.

We are aware that other abiotic and biotic variables, such as relativity humidity, rainfall and crop phenology, may affect the dynamics of the system. However, increasing the complexity of the model is most likely not necessarily a way to an optimized goal, as noted by Box^33^. We believe that the model presented here provides important information to enhance the reduction of sugarcane borer populations by optimizing the release strategy of the natural enemy. We assumed important aspects of the biology of both parasitoid and host, following the philosophy that models should be made as realistic and as simple as possible to achieve the goal^9^. In summary, the model presented here opens a wide field to be explored in augmentative biological control, when a new releasing strategy needs to be created or an old one needs to be updated.

### Supplementary Information

Supplementary Information.

## Data Availability

The computational code that supports the findings of this study is available from São Paulo Advanced Center for Biological Control (SPARCBio) but restrictions apply to the availability of this code which is used under license for the current study, and so is not publicly available. It is however available from the authors upon reasonable request and with permission of SPARCBio (FAPESP—Koppert, 2018/02317-5).
