# Supplementary material for: Optimizing the releasing strategy used for the biological control of the sugarcane borer Diatraea saccharalis by Trichogramma galloi with computer modeling and simulation
Source: Sci Rep. 2024 Apr 25;14:9535. doi: 10.1038/s41598-024-60146-y (PMC11045834; doi:10.1038/s41598-024-60146-y)
Supplement: Supplementary file 1 — Supplementary Information. [file 41598_2024_60146_MOESM1_ESM.docx]

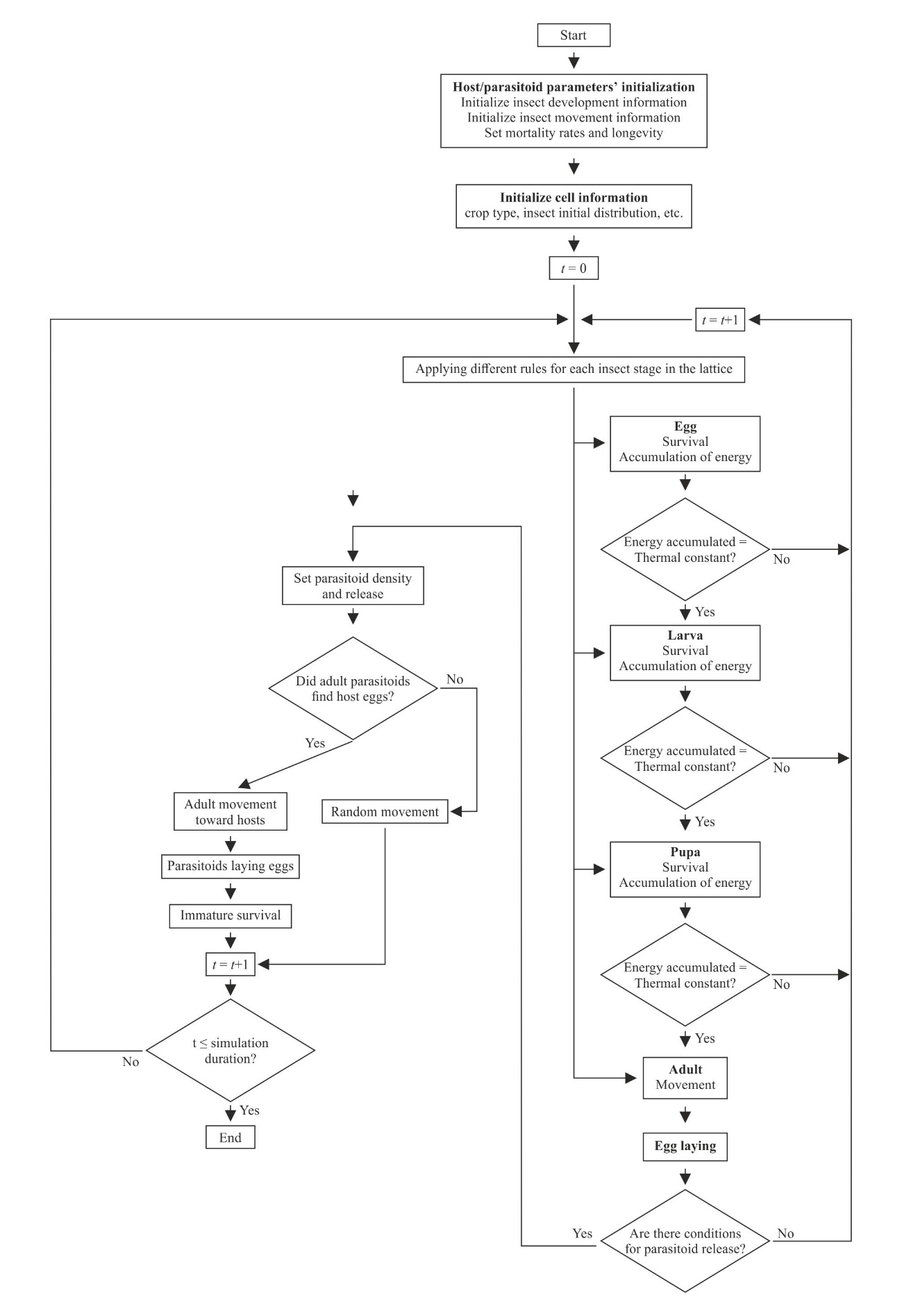


Supplementary Figure 1. Flowchart of the model structure for *Diatraea saccharalis* and *Trichogramma galloi*, describing the steps involved in the simulation
